# Sex differences in the association of physical activity levels and vitamin D with obesity, sarcopenia, and sarcopenic obesity: a cross-sectional study

**DOI:** 10.1186/s12877-022-03577-4

**Published:** 2022-11-24

**Authors:** Shuli Jia, Wanyu Zhao, Fengjuan Hu, Yunli Zhao, Meiling Ge, Xin Xia, Jirong Yue, Birong Dong

**Affiliations:** 1 National Clinical Research Center of Geriatrics, West China Hospital, Sichuan University, No. 37, Guo Xue Xiang Renmin Nan Lu, Chengdu, 610041 Sichuan China; 2 Center of Gerontology and Geriatrics, West China Hospital, Sichuan University, No. 37, Guo Xue Xiang Renmin Nan Lu, Chengdu, 610041 Sichuan China

**Keywords:** Vitamin D, Sarcopenia, Obesity, Sarcopenic obesity, Elderly, WCHAT

## Abstract

**Background:**

The relationship between vitamin D and sarcopenia was inconsistent between men and women. Physical activity (PA) may interact with vitamin D on sarcopenia. However, the sex-specific relationships of vitamin D, PA and sarcopenia have yet elucidated. We aimed to examine the sex differences in the relation between vitamin D status, PA levels, obesity and sarcopenia in community-dwelling middle-aged and older adults, as well as whether vitamin D status is a modifier in the relationship between PA and sarcopenia.

**Methods:**

The current study was a cross-sectional study based on the baseline survey of the West China Health and Aging Trend (WCHAT) study. A total of 3713 participants aged ≥ 50y were included in our study. Sarcopenia was defined according to the Asian Working Group for Sarcopenia (AWGS) 2019 consensus. Obesity was defined by body mass index (BMI) (≥ 28 kg/m2) and body fat mass percentage (≥ 60th percentile in each sex group). 25-hydroxyvitamin D was measured by chemiluminescent microparticle immunoassay and PA was evaluated by a validated China Leisure Time Physical Activity Questionnaire (CLTPAQ). Multinomial logistic regression was performed to examine the relationship between PA, vitamin D and sarcopenia and obesity.

**Results:**

Low PA was significantly associated with higher odds of sarcopenia in women only (OR = 1.70,95%CI:1.18,2.46, *p* < 0.01). Vitamin D deficiency was only associated with sarcopenia in men (OR = 1.85,95%CI: 1.27,2.69, *p* < 0.01). Low PA was significantly associated with obesity, sarcopenia, and sarcopenic obesity only in participants with serum 25(OH)D < 20 ng/ml.

**Conclusions:**

The role of vitamin D and PA in obesity and sarcopenia was different between men and women, and the relationship between PA and sarcopenia was modified by serum vitamin D status. These findings highlighted the need to supplement vitamin D in individuals with physical inactivity and provide different interventions strategies to sarcopenia in men and women.

**Trial registration:**

Clinical trial number: ChiCTR1800018895.

**Supplementary Information:**

The online version contains supplementary material available at 10.1186/s12877-022-03577-4.

## Background

Sarcopenia is an age-related skeletal muscle disorder defined as accelerated loss of muscle mass and function [1]. Sarcopenia is associated with multiple adverse outcomes such as falls, physical disability, frailty and premature death [1]. In parallel with the development of sarcopenia, the prevalence of obesity is also rising with aging [2]. The concurrent presence of both sarcopenia and obesity in the same individual is defined as sarcopenic obesity (SO), which is a better predictor of negative health outcomes in older than sarcopenia or obesity alone [2, 3].

Physical inactivity and vitamin D deficiency often co-exist in older adults, and they are both risk factors for sarcopenia and obesity [1, 4, 5]. Previous studies have reported the role of vitamin D in the development of sarcopenia [6]. Serum levels of vitamin D are independently related to the loss of muscle mass and strength [7], particular in men [8]. Vitamin D deficiency is frequently observed in obese [9]. Evidence from observational studies suggests that obesity is associated with low vitamin D status as a result of the inverse between serum 25(OH)D concentration and body fat mass [10]. To our knowledge, studies related to the associations of physical activity levels, vitamin D, and sarcopenic obesity are limited. There are differences in physical activity levels and vitamin D between males and females. Vitamin D deficiency or physical inactivity as risk factor for sarcopenia may not be equally important in men and women. Sex differences were often reported in the relationship between vitamin D and sarcopenia [11, 12], or in the effect of vitamin D supplementation on sarcopenia [13].

Physical inactivity and vitamin D deficiency may have synergistic effects on muscle degradation and exacerbate sarcopenia [14]. A previous study explored the interactive effect of vitamin D and physical activity on muscle mass and function through animal experiments and population surveys [14]. They concluded that the effect of vitamin D on sarcopenia depends on the level of physical activity in older adults [14]. Specifically, physical activity levels modified the relationship of vitamin D and sarcopenia. At present, the treatment of sarcopenia mainly focuses on physical activity and nutrition supplementation such as vitamin D [1]. A large number of clinical studies have investigated the effects of physical activity and vitamin D supplementation on muscle mass and strength singly or combinedly [13, 15–18]. However, there is still inconsistent in results. A network meta-analysis of randomized controlled trials found that combining vitamin D supplementation with protein supplementation and exercise can significantly increase grip strength, which indicates that adding vitamin D to a standard treatment protocol for sarcopenia may be helpful for regaining function [18]. Vitamin D status seems to be an important factor for intervention to sarcopenia in clinical studies.

Therefore, the major purpose of this study was to examine whether the role of physical activity and vitamin D in sarcopenia, obesity, and sarcopenic obesity was different between men and women. The secondary objective was to investigate the relationship of physical activity and sarcopenia by different vitamin D status to examine whether vitamin D status is a modifier in this relationship.

## Methods

### Study design and participants

Data used in the present study are from the baseline survey of West China Health and Aging Trend (WCHAT) study [19]. A total of 7536 community-dwelling participants aged ≥ 50 years were enrolled at baseline survey in July 2018. Face-to-face interviews were performed by trained interviewers. Information on demographics, health and chronic diseases, physical performance, anthropometric measurements, body composition, and fasting blood were collected. This study was approved by the Ethics Committee of West China Hospital, Sichuan University and conducted in accordance with the Declaration of Helsinki. This study was registered at the China Clinical Trial Center (Registration Number: ChiCTR1800018895). All participants signed the informed consent before participating.

After excluding 3474 participants who have missing data on grip strength, skeletal muscle mass index (SMI) and Short Physical Performance Battery (SPPB), 74 missing data on vitamin D, and 275 missing data on covariates, 3713 participants aged 50 years and older were included in the final analysis.

### Definitions of obesity, sarcopenia, and sarcopenic obesity

We define obesity based on two different obesity markers: a) body mass index (BMI) ≥ 28.0 kg/m2 [20]; b) body fat percentage (based on bioelectrical impedance analysis) ≥ 60th percentile of the weighted sample distribution, adjusting for sex (in our population, the cut-off values was 30.35% in male and 38.64% in female, respectively). Participants were defined as obese if they met either of these two criteria.

Sarcopenia was defined according to the Asian Working Group for Sarcopenia (AWGS) 2019 consensus [21], which included low muscle strength (handgrip strength: < 28 kg for male, < 18 kg for female), low muscle mass (Appendicular skeletal muscle mass index (ASMI, ASM/height2): bioelectrical impedance analysis (BIA): < 7.0 kg/m2 for male, 5.7 kg/m2 for female), and poor physical performance assessed by Short Physical Performance Battery (SPPB ≤ 9). Combination of low muscle mass and low muscle strength (or low physical performance) was defined as sarcopenia. Muscle mass was measured with a bioelectrical impedance analyzer (Inbody 770, BioSpace, Seoul, Korea), which is a simple, and precise method for assessment of skeletal muscle mass. Handgrip strength was measured with the dominant hand using a dynamometer (EH101; Camry, Zhongshan, China). Two tests were performed and the highest value was considered. We used SPPB to assess physical performance, which included standing balance test, 4-m walk time and 5-time chair stand test. The total score ranges from 0 to 12, and participants scoring 9 and less are considered low physical performance.

Participants were categorized into four groups: 1) Normal (N): neither obesity nor sarcopenia; 2) Obesity (O): only presence of obesity; 3) Sarcopenia (S): only presence of sarcopenia; 4) Sarcopenic obesity (SO): both presence of obesity and sarcopenia.

### Physical activity assessment

Self-reported physical activity (PA) was evaluated by a validated China Leisure Time Physical Activity Questionnaire (CLTPAQ) [22], which was a modified version of the Minnesota Leisure Time Physical Activity Questionnaire (MLTPAQ) [23] according to the Chinese lifestyles and cultural background. The CLTPAQ consists the frequency and duration of common activities in the last two weeks: walking, indoor housework (such as scrubbing the windows, or mopping the floor), outdoor housework (such as cleaning the yard (terrace), tidying up the garden, or shoveling snow), dancing and other regular activities (such as strength exercises, swimming, cycling, jogging, or tai chi). We calculated the energy consumption per week according to metabolic equivalents of task (MET) of each type of activity [24]. (Note: energy consumption (kcal /week) = MET * Times per week * Minutes per time * Weight(kg)/60). walking (4.0 MET), indoor housework (3.5 MET), outdoor housework (5.0 MET), dancing (4.5 MET), and other regular exercises such as swimming, jogging (5.0 MET)). Physical activity level was divided into two groups according to the 20th percentile of energy consumption per week, adjusting for sex. Moderate to high physical activity was defined as being ≥ the 20th percentile of energy consumption per week. Low physical activity was defined as being < the 20th percentile of energy consumption per week.

### Serum vitamin D measurement

Venous blood samples were collected after an overnight fast to measure biochemical parameters. Serum 25(OH)D concentration (ng/mL) was performed using chemiluminescent microparticle immunoassay (Abbott i2005R, USA). Participants were classified into two groups according to serum vitamin D concentration: vitamin D deficiency (25(OH)D < 20 ng/ml) and vitamin D insufficiency or sufficiency (25(OH)D ≥ 20 ng/ml). All laboratory evaluations were performed by trained clinical laboratory technicians in accordance with standard operating procedures in hospital laboratories.

### Covariates

Demographics and lifestyle data including age, sex, ethnicity, education (illiterate, primary school, middle school, high school and above), marital status (married, single/divorced/widowed), smoking history were collected. Self-reported diagnosed chronic diseases, including hypertension, diabetes mellitus, coronary heart diseases, chronic obstructive pulmonary diseases (COPD), arthritis, stroke, and tumor were also interviewed. We also included disability in activities of daily living (ADL disability). ADL disability was defined as having needs for assistance or difficulty in one or more of the ten items in Barthel Index [25]. Nutrition status was evaluated with the Mini Nutrition Assessment-Short form (MNA-SF) [26] with 8–11 points indicating risk of malnutrition and < 8 points indicating malnutrition.

### Statistical analysis

Statistical analysis was performed using Stata15.1 (Stata Corp, College Station, TX, USA). We investigated participants’ characteristics using descriptive statistics and appropriate statistical tests. For descriptive analysis, continuous variables are expressed as the mean ± standard deviation (SD), and categorical variables are expressed as number (%). Group differences were evaluated using the Student’s t-test for continuous variables and the chi-squared tests for categorical variables.

We used multinomial logistic regression to examine the relationship between PA levels (exposure, binary variable), vitamin D status (exposure, binary variable), and obesity or sarcopenia status (outcome, categorial variable). We also conducted sex subgroup analysis to explore the different role of PA and vitamin D in obesity and sarcopenia in men and women. Furthermore, we investigated the relationship of PA and obesity/sarcopenia at different vitamin D status to explore whether vitamin D could modify this relationship. All regression models were adjusted for age, sex, ethnicity, education, marital status, smoking history, common chronic diseases, ADL disability, and nutrition status. Two-sided *p* < 0.05 was considered statistically significant.

## Results

### Characteristics of participants

A total of 3713 participants aged 50 years and older were included our analysis. The mean age was 61.9 years (SD 8.0), and 42% were aged < 60 years (Table 1). For the whole sample, 1351 (36.4%) were men. The prevalence of obesity, sarcopenia and sarcopenic obesity were 37.8%, 12.6%, and 4.8%, respectively. The mean concentration of 25(OH)D was 19.2 ng/ml (SD 6.3), and 17.8% participants were identified as low PA. 59.3% participants had vitamin D deficiency (25(OH)D < 20 ng/ml). There were significant differences between male and female on demographic and some chronic diseases. Compared with men, women were younger (Table 1). The vitamin D concentration of men was significantly higher than women, while the prevalence of low PA in men was higher than that in women. Compared with women, men were more likely to be sarcopenia and sarcopenic obesity.

**Table 1:** Characteristics of participants according to sex (*N* = 3713)

**Characteristics**	**Total** **(*n* = 3713)**	**Male** **(*n* = 1351)**	**Female** **(*n* = 2362)**	***P*** **value**
Age(years), mean (SD)	61.9(8.0)	63.5(8.1)	61.0(7.8)	< 0.001
< 60y, n (%)	1528(41.2)	441(32.6)	1087(46.0)	< 0.001
≥ 60y, n (%)	2185(58.8)	910(67.4)	1275(54)	
Ethnicity, n (%)				< 0.001
Han	1628(43.8)	538(39.8)	1090(46.1)	
Qiang	936(25.2)	329(24.4)	607(25.7)	
Tibetan	931(25.1)	403(29.8)	528(22.4)	
Others	218(5.9)	81(6.0)	137(5.8)	
Education, n (%)				< 0.001
Illiterate	1068(28.8)	219(16.2)	849(35.9)	
Elementary	1274(34.3)	532(39.4)	742(31.4)	
Middle	832(22.4)	326(24.1)	506(21.4)	
High and above	539(14.5)	274(20.3)	265(11.2)	
Marital status, n (%)				< 0.001
Married	3184(84.7)	1229(91.0)	1921(81.3)	
Single/divorced/widowed	575(15.3)	122(9.0)	441(18.7)	
Smoking history, n (%)				< 0.001
Yes	657(17.7)	609(45.1)	48(2.0)	
No	3056(82.3)	742(54.9)	2314(98.0)	
Chronic diseases, n (%)				
Hypertention	930(25.1)	349(25.8)	581(24.6)	0.40
COPD	113(3.0)	16(1.2)	18(0.8)	0.19
Coronary heart disease	34(0.9)	47(3.5)	66(2.8)	0.24
Diabetes	264(7.1)	113(8.4)	151(6.4)	0.025
Arthritis	330(8.9)	102(7.5)	228(9.7)	0.030
Stroke	63(1.7)	27(2.0)	36(1.5)	0.28
Tumor	26(0.7)	4(0.3)	22(0.9)	0.026
Nutrition status, n (%)				0.074
Normal	3015(81.2)	1106(81.9)	1909(80.8)	
Risk of malnutrition	680(18.3)	243(18.0)	437(18.5)	
Malnutrition	18(0.5)	2(0.1)	16(0.7)	
ADL disability, n (%)	351(9.5)	128(9.5)	223(9.4)	0.97
Vitamin D (ng/ml), mean (SD)	19.2(6.3)	21.4(6.5)	18.0(5.8)	< 0.001
Vitamin D status, n (%)				< 0.001
< 20 ng/ml	2203(59.3)	609(45.1)	1594(67.5)	
≥ 20 ng/ml	1510(40.7)	742(54.9)	768(32.5)	
PA levels, n (%)				< 0.001
Moderate to high	3053(82.2)	1041(77.1)	2012(85.2)	
Low	660(17.8)	310(22.9)	350(14.8)	
Sarcopenia and Obesity, n (%)				< 0.001
N	1665(44.8)	583(43.2)	1082(45.8)	
O	1404(37.8)	485(35.9)	919(38.9)	
S	467(12.6)	190(14.1)	277(11.7)	
SO	177(4.8)	93(6.9)	84(3.6)	

*Abbreviations: ADL* Activity of daily living, *PA* Physical activity, *N* Normal, *O* Obesity, *S* Sarcopenia, *SO* Sarcopenic obesity

### Relationship of physical activity, vitamin D, with obesity, sarcopenia and sarcopenic obesity

Table 2 shows the results of multinomial logistic regressions of associations between PA, vitamin D and sarcopenia. In total sample, after adjusting for confounding variables, low PA was significantly associated with higher odds of sarcopenia (OR = 1.65, 95%CI: 1.25,2.18, *p* < 0.001). Vitamin D deficiency was significantly associated with obesity, sarcopenia, and sarcopenic obesity (OR = 1.57, 95%CI: 1.34,1.84, *p* < 0.001, OR = 1.31, 95%CI: 1.03,1.66, *p* = 0.029, and OR = 1.88, 95%CI: 1.32,2.67, *p* < 0.001, respectively). In subgroup analysis, low PA was significantly associated with sarcopenia only in women. Vitamin D deficiency was independently associated with obesity, sarcopenia, and sarcopenic obesity in men, while significant association was only observed for obesity and sarcopenic obesity in women.

**Table 2:** Associations between physical activity, vitamin D and obesity/sarcopenia in total sample and sex subgroup

**Groups**	**ORs(95%CI)**		
	**O vs N**	**S vs N**	**SO vs N**
**Total sample (*n* = 3713)**			
PA levels			
Moderate to high	1(reference)	1(reference)	1(reference)
Low	1.19(0.97,1.47)	1.65(1.25,2.18) ***	1.48(1.00,2.19)
Vitamin D			
≥ 20 ng/ml	1(reference)	1(reference)	1(reference)
< 20 ng/ml	1.57(1.34,1.84) ***	1.31(1.03,1.66) *	1.88(1.32,2.67) ***
**Male (*n* = 1351)**			
PA levels			
Moderate to high	1(reference)	1(reference)	1(reference)
Low	1.38(1.00,1.91)	1.56(1.00,2.42)	1.54(0.89,2.68)
Vitamin D			
≥ 20 ng/ml	1(reference)	1(reference)	1(reference)
< 20 ng/ml	1.75(1.34,2.28) ***	1.85(1.27,2.69) **	2.05(1.28,3.30) **
**Female (*n* = 2362)**			
PA levels			
Moderate to high	1(reference)	1(reference)	1(reference)
Low	1.05(0.79,1.38)	1.70(1.18,2.46) **	1.58(0.90,2.80)
Vitamin D			
≥ 20 ng/ml	1(reference)	1(reference)	1(reference)
< 20 ng/ml	1.47(1.20,1.79) ***	1.06(0.77,1.45)	1.93(1.11,3.37) *

In total sample adjusted age, sex, ethnicity, education, marital status, smoking, ADL disability, chronic diseases (hypertention, coronary heart diseases, COPD, diabetes, stroke, arthritis, tumor), nutrition status, physical activity, and vitamin D status. In sex subgroups, adjusted all variables in total sample analysis except for sex

*Abbreviations: ORs* Odds ratios, *CI* Confident interval, *N* Normal, *O* Obesity, *S* Sarcopenia, *SO* Sarcopenic obesity, *PA* Physical activity

^*^p < 0.05, **p < 0.01, ***p < 0.001

### Subgroup analysis of vitamin D status in the relationship of physical activity with obesity, sarcopenia, and sarcopenic obesity

Table 3 presents the relationships of physical activity levels and sarcopenia according to vitamin D status. Low PA was only significantly associated with obesity, sarcopenia and sarcopenic obesity in participants who had vitamin D deficiency (OR = 1.38, *p* < 0.05, OR = 1.96, *p* < 0.001 and OR = 1.85, *p* < 0.05, respectively).

**Table 3:** Association between physical activity and obesity/sarcopenia by vitamin D status

**Vitamin D subgroups**	**ORs(95%CI)**		
	**O vs N**	**S vs N**	**SO v N**
**Vitamin D < 20 ng/ml (*n* = 2203) **^a^			
Moderate to high	1(reference)	1(reference)	1(reference)
Low	1.38(1.04,1.83) *	1.96(1.34,2.85) ***	1.85(1.14,3.01) *
**Vitamin D** ≥ **20 ng/ml (*n* = 1510) **^a^			
Moderate to high	1(reference)	1(reference)	1(reference)
Low	0.98(0.71,1.35)	1.41(0.91,2.15)	1.07(0.53,2.13)

*Abbreviations: ORs* Odds ratios, *CI* Confident interval, *N* Normal, *O* Obesity, *S* Sarcopenia, *SO* Sarcopenic obesity

^a^ Adjusted age, sex, ethnicity, education, marital status, smoking, ADL disability, chronic diseases (hypertention, coronary heart diseases, COPD, diabetes, stroke, arthritis, tumor), nutrition status

^*^p < 0.05, **p < 0.01, ***p < 0.001

## Discussion

The present study adds evidence to the sex-specific relationships between vitamin D status, PA levels, sarcopenia, obesity and sarcopenic obesity among middle aged and older people in west China. Vitamin D deficiency, defined as total 25(OH)D < 20 ng/ml, was found to be associated with sarcopenia only in men, and low PA was found to be associated with greater odds of sarcopenia only in women. Vitamin D deficiency was independently associated with obesity and sarcopenic obesity both in men and women. We also found vitamin D status modified the association between PA and sarcopenia. Specifically, among participants with 25(OH)D < 20 ng/ml, low PA was associated with increased risk of sarcopenia, whereas there was no significant association among those with 25(OH)D ≥ 20 ng/ml.

Consistent with previous studies, our study confirmed that physical inactivity and vitamin D deficiency were associated with sarcopenia [27, 28]. Additionally, we found that the role of vitamin D and PA in sarcopenia was not equally important in men and women. Previous studies investigating the relation between vitamin D and sarcopenia have yielded conflicting results. In line with a previous study conducted by Spira et al. [12], vitamin D deficiency was significantly associated with sarcopenia only in men. In contrast, Park et al. [11] found a positive association of vitamin D deficiency and sarcopenia in ≥ 50 years old women, but not men [11]. These inconsistent results may be explained by different population and definitions of sarcopenia. Differences between men and women in many characteristics may be potential confounders affecting this relationship. For example, different rates of decline of hormones such as testosterone and estrogen in different life stages in men and women [29]. The hormonal changes of female menopause may outweigh the effect of a low vitamin D level on muscle mass. For men, the decline of testosterone usually occurs at an older age, therefore, a low vitamin D level might be more crucial concerning the onset of sarcopenia [12, 29]. Furthermore, vitamin D is inversely associated with obesity and their relationship may be bi-directional [30]. Women had higher total fat mass than men, which may lead to sequestration of the fat-soluble vitamin D in fat tissue and therefore decreased bioavailability of vitamin D3 [31]. This may be one reason why women had a lower 25(OH)D concentration than men. Therefore, fat mass likely might be a moderator and mediator of the relationship between vitamin D deficiency and sarcopenia.

Compared with the role of vitamin D in sarcopenia development in men, physical inactivity was the main contributor to developing sarcopenia in women. Previous studies have found that low PA was significantly associated with sarcopenia [32, 33]. In our results, low PA was found to be associated with sarcopenia only in women. A meta-analysis indicated that PA can significantly reduce the odds of both males and females suffering from sarcopenia (OR = 0.46, 95%CI: 0.37,0.58, and OR = 0.65, 95%CI: 0.52,0.81, respectively) [33]. We did not find a positive results in men, which may be explained by a relatively small sample in men and different methods of assessing PA. Although the definition of low physical activity was adjusted for sex in the present study. Physical activity in men is usually higher than that of women. Additionally, other factors may interact with physical activity, thus weakening the role of physical activity in sarcopenia.

Other possible factors cannot be completely ruled out, and more studies are still needed to further prove.

Low serum vitamin D was found to be associated with obesity and sarcopenic obesity regardless of sex. The inverse association between vitamin D and obesity has been established in the literature [10]. A large meta-analysis has shown that BMI-related genetic variants were associated with both BMI and vitamin D levels, while vitamin D-related genetic variants were only associated with vitamin D status but not BMI, which support the hypothesis that obesity may lead to low vitamin D level [30]. This result indicated that weight or percentage of fat mass loss may increase serum 25(OH)D [30].

Serum vitamin D status was an important modifier in the relationship between PA and sarcopenia. As shown in our results, participants with low PA had higher odds of sarcopenia and sarcopenic obesity when participants had vitamin D deficiency. However, low PA was not significantly associated with sarcopenia and sarcopenic obesity among participants with higher concentration of vitamin D (25(OH)D ≥ 20 ng/ml). Another cross-sectional study conducted by Yang et al. found that vitamin D and PA had an interactive effect on sarcopenia. The effects of vitamin D on sarcopenia may be affected by the level of PA, and the effects of PA may also be affected by vitamin D levels [14]. Yang et al. also found that activity limitation reduced the muscle fiber cross-sectional area and muscle weight in mice, and vitamin D deficiency accelerated the decrease in muscle weight, muscle fiber CSA, and grip strength. In addition, vitamin D supplementation may inhibit the grip strength reduction induced by limited activity [14]. These results may explain the controversial effects of vitamin D or exercise training on muscle mass and muscle function in clinical trials. The results of vitamin D supplementation or exercise therapy may be affected by differences in baseline PA levels and serum 25(OH)D levels in older adults. These findings indicated that participants with inactivity were important population to screen serum vitamin D status. And for them, maintaining sufficient vitamin D may reduce the risk of developing sarcopenia and sarcopenic obesity.

Our study had some strengths. First, WCHAT is a relatively large population-based study, and complete information on physical and health conditions was collected through standardized and structured interviews. Second, diagnosis of sarcopenia was based on the AWGS 2019 consensus, and definition of obesity was based on the combination of BMI and fat mass percentage assessed by BIA, which were more accurate and standard. However, several limitations should be considered in our current study when interpreting the results. First, cross-sectional analyses cannot elucidate any determination of the temporal nature of the associations between variables, for which longitudinal studies are required. Second, PA evaluation was based on self-reporting not accelerometer-measured physical activities, so potential recall bias may exist. Finally, our study population were from multiple ethnic groups. Although we adjusted for ethnicity in the regression models, the differences in vitamin D levels and PA habits between ethnic groups and whether these differences influence the relationships of them are not clear.

This study has several implications for prevention and intervention to sarcopenia in older people. Firstly, it is recommended that simultaneous screening for vitamin D levels and evaluating levels of PA in older adults. Older adults should strive to avoid both physical inactivity and vitamin D deficiency. Secondly, supplementing vitamin D may help reduce the risk of sarcopenia in individuals with physical inactivity or physical limitations. Thirdly, women are encouraged to increase PA, while men should maintain adequate vitamin D levels. In the future clinical trials for sarcopenia, the intervention strategies for men and women should be different or individualized.

## Conclusion

The role of vitamin D and PA in sarcopenia was different between men and women, and the relationship between PA and sarcopenia modified by serum vitamin D status. These findings highlighted the need to supplement vitamin D in individuals with physical inactivity and provide different interventions strategies to sarcopenia in men and women.

## Supplementary Information

**Additional file 1: Table S1.** Associations between physical activity, vitamin D and obesity/sarcopenia according to age group. **Table S2.** Vitamin D levels of the total sample and according to physical activity level.

## Data Availability

The datasets used and/or analyzed during the current study available from the corresponding author on reasonable request.

## Abbreviations

AWGS: Asian Working Group for Sarcopenia
ASMI: Appendicular skeletal muscle mass index
ADL: Activities of daily living
BMI: Body mass index
BIA: Bioelectrical impedance analysis
CLTPAQ: China Leisure Time Physical Activity Questionnaire
COPD: Chronic obstructive pulmonary diseases
CI: Confidence interval
MLTPAQ: Minnesota Leisure Time Physical Activity Questionnaire
MNA-SF: Mini Nutrition Assessment-Short form
OR: Odds ratio
PA: Physical activity
SD: Standard deviations
SPPB: Short Physical Performance Battery
SMI: Skeletal muscle mass index
SO: Sarcopenic obesity
WCHAT: West China Health and Aging Trend
