# Supplementary material for: Sex differences in the association of physical activity levels and vitamin D with obesity, sarcopenia, and sarcopenic obesity: a cross-sectional study
Source: BMC Geriatr. 2022 Nov 24;22:898. doi: 10.1186/s12877-022-03577-4 (PMC9701059; doi:10.1186/s12877-022-03577-4)
Supplement: Supplementary file 1 — Additional file 1: Table S1. Associations between physical activity, vitamin D and obesity/sarcopenia according to age group. Table S2. Vitamin D levels of the total sample and according to physical activity level. [file 12877_2022_3577_MOESM1_ESM.docx]

**Table S1. Associations between physical activity, vitamin D and obesity/sarcopenia according to age group.**

| **Groups** | **ORs(95%CI)** | | |
| --- | --- | --- | --- |
|  | **O vs N** | **S vs N** | **SO vs N** |
| **Age <60 (n=1528)** | | | |
| PA levels |  |  |  |
| Moderate to high | 1(reference) | 1(reference) | 1(reference) |
| Low | 1.08(0.80,1.48) | 2.14(1.25,3.65) ** | 1.02(0.39,2.65) |
| Vitamin D |  |  |  |
| ≥20ng/ml | 1(reference) | 1(reference) | 1(reference) |
| <20ng/ml | 1.13(0.90,1.43) | 0.84(0.52,1.35) | 2.23(0.98,5.09) |
| **Age ≥60 (n=2185)** | | | |
| PA levels |  |  |  |
| Moderate to high | 1(reference) | 1(reference) | 1(reference) |
| Low | 1.24(0.93,1.66) | 1.62(1.16,2.25) ****** | 1.71(1.10,2.67) * |
| Vitamin D |  |  |  |
| ≥20ng/ml | 1(reference) | 1(reference) | 1(reference) |
| <20ng/ml | 2.17(1.74,2.71) ******* | 1.77(1.34,2.35) *** | 2.22(1.49,3.29) ******* |

Abbreviations: ORs, odds ratios; CI, confident interval; N, normal; O, obesity; S, sarcopenia; SO, sarcopenic obesity; PA, physical activity.

In total sample adjusted age, sex, ethnicity, education, marital status, smoking, ADL disability, chronic diseases (hypertention, coronary heart diseases, COPD, diabetes, stroke, arthritis, tumor), nutrition status, physical activity, and vitamin D status. In sex subgroups, adjusted all variables in total sample analysis except for sex.

**p*<0.05, ***p*<0.01, ****p* <0.001

**Table S2. Vitamin D levels of the total sample and according to physical activity level.**

| **Vitamin D (ng/ml)** | **Total**  **(N=3713)** | **Moderate to high PA**  **(N=3053)** | **Low PA**  **(N=660)** | **P value** |
| --- | --- | --- | --- | --- |
| Vitamin D, mean± SD | 19.2±6.3 | 19.2±6.2 | 19.3±6.6 | 0.71 |
| Vitamin D, median (IQR) | 18.6(14.9,23.0) | 18.5 (14.9, 22.9) | 18.8(14.5,23.6) | 0.74 |
| <10, n (%) | 155 (4.2) | 124 (4.1) | 31 (4.7) | 0.39 |
| 10-20, n (%) | 2048 (55.2) | 1701 (55.7) | 347 (52.6) |  |
| 20-30, n (%) | 1307 (35.2) | 1067 (34.9) | 240 (36.4) |  |
| ≥30, n (%) | 203 (5.5) | 161 (5.3) | 42 (6.4) |  |

PA physical activity
